# Assessment of knowledge and awareness of stroke among the Syrian population: unveiling the current landscape in Syria through the first nationally representative study

**DOI:** 10.1038/s41598-024-64935-3

**Published:** 2024-07-04

**Authors:** Zelal Kharaba, Yassen Alfoteih, Feras Jirjees, Mohammad Albarbandi, Zainab Hussain, Hala Al Obaidi, Sara Mansour, Munazza Ahmed, Eyman Eltayib, Muna Barakat, Husam A. AlSalamat, Reem Alzayer, Sami El Khatib, Safwan Al-Mohammad, Souheil Hallit, Diana Malaeb, Hassan Hosseini

**Affiliations:** 1 Department of Clinical Pharmacy, College of Pharmacy, Al Ain University, 64141 Abu Dhabi, United Arab Emirates; 2 Faculty of Medical Sciences, Newcastle University, Newcastle upon Tyne, NE2 4HH UK; 3 Department of Pharmacy Practice and Pharmacotherapeutics, College of Pharmacy, University of Sharjah, Sharjah, United Arab Emirates; 4 College of Dental Surgery, City University Ajman, 18484 Ajman, United Arab Emirates; 5 College of General Education, City University Ajman, 18484 Ajman, United Arab Emirates; 6 Department of Neurosurgery, Ibn Al-Nafees Hospital, Damascus, Syria; 7 Department of Neurosurgery, Damascus Hospital, Damascus, Syria; 8 Department of Biology, College of Science, University of Baghdad, Baghdad, Iraq; 9 School of Pharmacy, Queen’s University, Belfast, UK; 10 School of Pharmacy, Lebanese International University, Beirut, Lebanon; 11 College of Pharmacy, Al Jouf University, Jouf, Saudi Arabia; 12 Department of Clinical Pharmacy and Therapeutics, School of Pharmacy, Applied Science Private University, Amman, Jordan; 13 Department of Basic Medical Sciences, Faculty of Medicine, Al-Balqa Applied University, Al-Salt, 19117 Jordan; 14 Department of Biopharmaceutics and Clinical Pharmacy, School of Pharmacy, University of Jordan, Amman, Jordan; 15 Clinical Pharmacy Practice, Mohammed Al-Mana College for Medical Sciences, Dammam, Saudi Arabia; 16 Department of Biomedical Sciences, Lebanese International University, Bekaa, Lebanon; 17 Center for Applied Mathematics and Bioinformatics (CAMB), Gulf University for Science and Technology, Mubarak Al-Abdullah, Kuwait; 18 Neurosurgery Praxis, 06886 Wittenberg, Germany; 19 School of Medicine and Medical Sciences, Holy Spirit University of Kaslik, P.O Box 446, Jounieh, Lebanon; 20 Department of Psychology, College of Humanities, Effat University, 21478 Jeddah, Saudi Arabia; 21 Applied Science Research Center, Applied Science Private University, Amman, Jordan; 22 College of Pharmacy, Gulf Medical University, Ajman, United Arab Emirates; 23 UPEC-University Paris-Est, Creteil, France; 24 RAMSAY SANTÉ, HPPE, Champigny sur Marne, France

**Keywords:** Stroke, Knowledge, Awareness, Syria, National study, Stroke, Risk factors

## Abstract

Stroke is a global public health concern, contributing to high rates of morbidity and mortality. In Syria, the current conflict and associated challenges have had a profound impact on healthcare infrastructure, including education and awareness programs related to stroke. An essential aspect of preventing stroke is the awareness of individuals. The study aimed to investigate factors associated with knowledge and awareness of stroke among Syrian people. A cross-sectional national representative study was conducted in Syria. The questionnaire was created on Google form and disseminated as a link through online platform social media like Facebook, WhatsApp, and Twitter. The population of the study was divided using proportionate random sampling into the 14 governorates. A random sample was selected from each area. The STROBE reporting guideline for cross-sectional studies was followed. Logistic regression analysis was performed to identify the factors associated with poor knowledge of stroke. A total of 1013 Syrian adults participated in the study. With more than half of them were females (53.5%) and employed (55.6%). Significant associations were found between ability to identify at least one correct risk factor and employability status (*p* = 0.029), single group (*p* = 0.036) and smokers (*p* < 0.001). In addition, significant associations were found between identifying at least one correct stroke symptom and smokers (*p* < 0.001) and no-obese people (*p* = 0.048). Furthermore, younger age group (below 30 years) were significantly able to list at least one correct stroke consequence compared to the older age groups (*p* = 0.025). Moreover, a significantly higher number of smokers compared to non-smokers correctly identified at least one stroke consequence (*p* = 0.019). The study revealed that there is a relatively weak understanding of the preventable nature of stroke among Syrian population. The overall awareness is still inadequate and varies depending on lifestyle factors and employment status.

## Introduction

Stroke is a significant public health concern worldwide, contributing to high rates of morbidity and mortality^1^. Adequate knowledge and awareness of stroke are crucial for timely recognition of symptoms, prompt medical intervention, and prevention strategies. In 2019, the Institute of Health and Evaluation published a report stating that stroke is the third most common cause of mortality and disability and the second leading cause of death globally^2^. Additionally, compared to industrialized countries, the prevalence of stroke has increased more recently in many developing nations^3,4^. For instance, stroke has been recorded as a leading cause of disability and death in the Middle Eastern countries in the past three decades^2,5^.

In certain countries, such as Syria, the ongoing conflict and its associated challenges have had a profound impact on healthcare infrastructure, including education and awareness programs related to non-communicable diseases^6^. The Syrian population has been grappling with the devastating consequences of conflict for over a decade. Amidst the chaos and disruption caused by the conflict, the focus on non-communicable diseases, including stroke, has often been overshadowed. As a result, the lack of awareness and knowledge about stroke among the Syrian population has become a critical issue. Limited access to healthcare services and resources, the destruction of medical facilities, and the displacement of individuals have hampered the dissemination of essential information about stroke. Moreover, the economic hardships faced by Syrians people have further exacerbated the situation, making it challenging to allocate resources to stroke awareness campaigns and educational initiatives^6^.

To reduce the risk of stroke, primary cerebrovascular accident prevention is crucial which is accomplished through a variety of techniques focused on identifying related risk factors, implementing preventive techniques, and raising patient awareness^7–11^. The best preventive strategies include community-based educational initiatives through an evaluation stroke awareness and the variables that can trigger its occurrence^12–15^. Enhancing patient knowledge improves quality of life, and minimizes the work burden on the medical staff by decreasing admission rate^12^. It is worth mentioning that according to National Institute of Neurological 80% of stroke can be prevented through proper measures and activities directed towards decreasing the development of stroke and halting further progression of complications Enhancing patient knowledge not only improves the quality of life but may also reduce the risk of recurrent strokes, leading to fewer hospitalizations and a lower burden on the medical staff in the long term^16^. Moreover, it has been shown that in developing nations, there is always a concern about the general public awareness regarding the risk factors associated with stroke that increase morbidity and mortality^10,17–20^.

The lack of awareness regarding stroke symptoms, risk factors, and preventive measures has far-reaching consequences for the Syrian population^6^. Delayed recognition of stroke symptoms can lead to delayed medical intervention, resulting in increased morbidity and mortality rates. Addressing the knowledge gap and enhancing stroke awareness among the Syrian population is a vital step towards reducing the impact of stroke on individuals and the healthcare system. Therefore, it is crucial to assess public knowledge related to lifestyle, behavior, socioeconomic status, educational attainment, and smoking habits^21–23^. Both socioeconomic status and educational level are considered influential factors on stroke knowledge level where studies showed better stroke knowledge both with higher socioeconomic and educational levels. Research showed that individuals with higher educational level attainment and income are more likely able to seek knowledge to prompt better medical care^24,25^.

Comprehensive research focusing on the factors associated with knowledge and awareness of stroke among Syrians is essential to design targeted interventions and educational campaigns tailored to the unique challenges faced by the population.

The aim of the study was to investigate factors associated with knowledge and awareness of stroke among Syrians after more than a decade of conflict. By understanding these factors, we can develop evidence-based strategies to improve stroke education and awareness, considering the specific context and challenges of the Syrian population.

## Methods

### Study design and setting

The Strengthening the Reporting of Observational Studies in Epidemiology (STROBE) reporting guideline for cross-sectional studies was adopted and followed as a protocol for conducting this study^26^. A cross-sectional survey was conducted aiming at the general population in all major cities in Syria (Damascus, Rif Dimashq, Aleppo, Daraa, Deir ez-Zor, Hama, AlHasakah, Qamishli, Homs, Idlib, Latakia, Quneitra, AlRaqqah, As-Suwayda, and Tartous). The data were collected during the period from September 2021 to March 2022. A nationally representative sample of Syrian people was questioned using a structured self-administered questionnaire.

### Sampling technique

The survey was an open survey administered through online platform and was distributed by Google form through popular social media platforms like Facebook, WhatsApp, and Twitter. Participants were informed at the beginning of the survey about the eligibility criteria, including the requirement to be located in Syria. We also provided clear instructions for participants to answer the survey only if they meet the eligibility criteria. he population of the study was divided using proportionate random sampling into the 14 governorates. Each governorate was stratified geographically into rural and urban areas. A random sample was selected from each area. Chain-referral sampling was used in the distribution phase, where respondents were asked to send the questionnaire to their family members, friends, and colleagues.

### Sample size calculation

Syria's estimated population is approximately 22 million people (11,046,621 female (49.69%), 11,067,684 male (50.31%). The age groups range between adults (18 years) of more than 62%^27,28^. A statistical power analysis was conducted for sample size calculation using Raosoft Sample size calculator, with an accepted margin of error 5%, confidence level of 95%, population size of 22 million and 50% response of distribution. The sample size was calculated using Raosoft online software available^29^. The minimum sample size was found to be 385.

The study population size is described in Table 1. The Syrian Governorate was divided into three main areas; (i) Central and South Area that contains (Damascus, Rif Dimashq/Quneitra, As-Suwayda, Daraa, and Homs), (ii) Eastern Area that contains (Deir ez-Zor, AlHasakah, Qamishli, and AlRaqqah) and (iii) North-Western and central Area that contain (Aleppo and Hama). Each area was further stratified into districts based on population density. Two districts from each governorate were chosen randomly, and then, a random sample of adults was approached from each district. During data collection, 341 of participants (33.7%) were enrolled from Central and South Area based on its proportion of adults from the total number of the study population as shown in Table 1. Similarly, 335 participants (33.0%) were recruited from Eastern Area, and 337 participants (33.3%) were recruited from North-Western Area.

**Table 1:** Calculation of proportionate sample distribution in Syria (n = 1013).

	Governorate	Population	Total number of populations per Area	%	Sample size in the study
Central and South Area	Damascus	1,711,000	17.871.689	80.8%	341 (33.7%)
	Rif Dimashq/Quneitra	2,273,074			
	As-Suwayda	313,231			
	Daraa	998,000			
	Homs	652,609			
	Other areas	11,923,775			
Eastern Area	Deir ez-Zor	211,857	804,736	3.6%	335 (33.0%)
	AlHasakah	188,160			
	Qamishli	184,231			
	ArRaqqah	220,488			
North-Western Area	Aleppo	2,132,100	3.437.880	15.5%	337 (33.3%)
	Hama	312,994			
	Idlib	165,000			
	Latakia	383,786			
	Tartous	444,000			
Total		22.114.305	22.114.305	100%	1013

### Study eligibility

Inclusion criteria of the study included individuals at least 18 years old, Syrian and living in Syria, and an Arabic speaker. Exclusion criteria are adults who were mentally incompetent, critically ill or suffered from stroke. A screening questions at the beginning of the survey was added to identify participants who may not meet the inclusion criteria. We provided clear instructions for participants to refrain from taking the survey if they did not meet the eligibility criteria. While we recognize the limitations of self-reported data in assessing these conditions, we aimed to minimize the inclusion of participants who did not meet the specified criteria. A total of 1350 participants answered the questionnaire. Of those, 337 people were excluded for not meeting the inclusion criteria, or did not answer all the questions. This gave us a final sample size of 1013 participants.

### Study tools

The questionnaire utilized in this study was adapted from previously published studies in the literature review^17,19^. The structure of the questionnaire closely resembled the one used in the study conducted in Jordan, covering various aspects of stroke knowledge, such as symptoms, risk factors, early warning signs, and complications^19^. However, modifications were made to account for sociodemographic factors, economic status, and residence area. Participants were required to complete the questionnaire independently, without assistance from investigators, to minimize potential response bias.

The questionnaire consisted of two sections. The first section focused on gathering sociodemographic information, including age, marital status, smoking status, employment status, monthly income, residence, educational level, and self-reported medical history, such as hypertension, diabetes mellitus, and dyslipidemia. The second section of the questionnaire assessed participants' general knowledge related to stroke including early warning signs of stroke. The participants were also queried about their awareness of potential consequences of stroke, Furthermore, several questions explored participants' attitudes and reactions towards stroke patients, curiosity and self-assessment, and the sources from which they acquired knowledge about stroke.

For each correct response, participants were awarded one point. Missing answers were not considered, and in some instances, multiple answers were allowed, resulting in a total score that could exceed the number of questions. The survey took approximately 10–15 min to complete. Responses of participants were kept anonymous and confidential. Participants had the choice and the right to withdraw from filling the questionnaire or ignore answering any question without justification.

### Validation and reliability testing of the study questionnaire

The study instrument was a researcher-administered questionnaire developed to serve the purpose of this research. The study tool was tailored to Syrian population after extensive literature review of previous studies that assessed different factors associated with knowledge and awareness of stroke worldwide and in the region^17,19,30–32^. The Arabic-translated version of the questionnaire was used and validated by 3-bilingual linguistics using forward and backward translation technique^33^ to be suitable for researching the Syrian population.

In addition, another validation test was conducted for the edited version of the questionnaire. A draft of the questionnaire was prepared and sent to a panel for content validation of a questionnaire which considered the length and conciseness, language, clarity, time, bias, and appropriateness of questions.

The primary investigator extended invitations to four experts and professors in epidemiology and clinical sciences in Al Ain University (UAE), University of Sharjah (UAE), Applied Science Private University (Jordan), and Lebanese International University (Lebanon) to test the content validity of the questionnaire. Additionally, four individuals from the public, healthy adults from both genders and a non medical background, were also invited to attend a virtual meeting. The purpose of the meeting was to validate the content of the study questionnaire. Each invitee was asked to evaluate every item in the questionnaire using a scale ranging from 1 to 10. The evaluation criteria included clarity, relevance, appropriateness, question length, and the time required to complete the questionnaire. The average scores (± SD) for clarity, relevance, appropriateness, question length, and time required were 8.4 ± 1.5, 8.4 ± 1.5, 8.7 ± 1.2, 9.2 ± 0.7, and 8.5 ± 1.4, respectively.

To ensure the reliability of the study questionnaire, the suggestions for modifications and amendments provided by the invitees were carefully considered by the research team. Subsequently, a pilot test was conducted using the validated version of the questionnaire. The pilot test involved 28 participants who were instructed to complete the survey and identify any questions or wording that could potentially hinder their understanding of the questionnaire. The responses obtained were imported into the SPSS and the internal consistency of the questionnaire items was calculated.

### Pilot study

It was crucial to make sure the right information was gathered for the pilot test while creating the final questionnaire. Pilot testing was intended to find such elements that respondents found unclear. Twenty-eight respondents (two from each governance) who met the inclusion criteria were invited to complete the questionnaire as part of the tool's pilot phase. The data collected was examined, and the subjects' feedback about potential challenges encountered was obtained. The Cronbach alpha (α) coefficient to measure the reliability of the survey was greater than 0.8.

### Ethical consideration

The study protocol was approved by the Research Ethics Committee from the Research Ethics Committee of Al Ain University, UAE (REC_AAU_September 2021_B3). The committee approved the research, and we confirm that all research was performed in accordance with the relevant regulations. Informed consent was obtained from all participants involved in the study.

### Statistical analysis

Data collected were organized and analyzed using the Statistical Package for Social Sciences (SPSS) version 26.0. Continuous variables were presented as mean ± standard deviation and 95% confidence interval (CI). Categorical and ordinal variables were shown as frequencies (n) and percentages (%). Binary logistic regression was performed to determine the factors associated with the ability to spontaneously answer at least one or more stroke risk factors, one or more warning signs, one or more consequences, and seeking an emergency room as soon as stroke develops based on previous data. Variables with a p < 0.2 in the bivariate analysis were included in the regression analysis. Results were presented as odds ratios (OR) and 95% CI. Statistical tests were two-tailed and reported statistically significant at p < 0.05.

### Ethics approval

The study received the required ethical approvals from the Research Ethics Committee (REC) (AAU-REC-B3, Feb 2021). The study also obtained the IRB approval (IRB: FU-27412: 2022) from Al Furat University in Syria.

### Informed consent

Informed consent was obtained from all participants involved in the study; written informed consent has been obtained from the participant(s) to publish this paper.

## Results

### Characteristics of study participants

A total of 1013 individuals responded to the questionnaire and were enrolled in this study. More than half of the sample (53.5%) were females and employed (55.6%). More than one-third of the participants (38.7%) were below 30 years and were single (42.2%). The majority lived in urban areas (78.8%), had low monthly income (62.3%), and were smokers (67.0%). Moreover, most of them achieved a higher education qualification (university level) (65.0%). The most frequently reported chronic diseases of the respondents were hypertension (28.3%), and dyslipidemia (21.2%).

Almost all participants (96.8%) had heard about stroke before. Few described a family history of stroke (18.2%) while a considerable proportion (79.9%) personally knew someone suffering from stroke. Sociodemographic characteristics, past medical history, and general stroke knowledge are presented in Table 2.

**Table 2:** Socio-demographics, past medical history, and familiarity with stroke of respondents (n = 1013).

Socio-demographic characteristics		Frequency (%)
**Gender**	Male	471 (46.5%)
	Female	542 (53.5%)
**Age groups (years)**	< 30	392 (38.7%)
	30–49	369 (36.4%)
	> 50	252 (24.9%)
**Residence area**	Urban	798 (78.8%)
	Rural	215 (21.2%)
**Marital status**	Single	427 (42.2%)
	Married	282 (27.8%)
	Divorced	126 (12.4%)
	Widowed	178 (17.6%)
**Education level**	School	355 (35.0%)
	University	658 (65.0%)
**Employment status**	Unemployed	450 (44.4%)
	Employed	563 (55.6%)
**Income level (**Syrian Lira)	Low (< 70,000)	631 (62.3%)
	Medium (70,000—100,000)	226 (22.3%)
	High (> 100,000)	156 (15.4%)
**Smoking status**	Yes	679 (67.0%)
**Past medical history**	Hypertension	287 (28.3%)
	Diabetes Mellitus	97 (9.6%)
	Dyslipidemia	215 (21.2%)
	Arrhythmia	201 (19.8%)
	Kidney disease	86 (8.5%)
	Peptic ulcer	329 (32.5%)
	Depression	162 (16.0%)
	Obesity	100 (9.9%)
**Familiarity with stroke**	Ever heard of stroke	981 (96.8%)
	History of stroke in the family	184 (18.2%)
	Personally know someone with stroke	809 (79.9%)

## Respondents’ general knowledge about stroke

Analysis of correct responses showed that 98.3%, 97.9%, and 98.6% of the participants were able to correctly identify at least one established stroke risk factor, symptom, and consequence, respectively. However, only 24.3% of the participants identified all the risk factors, 25.2% of the participants recognized all the symptoms, and 31.8% of the participants stated all possible consequences of stroke (Table 3).

**Table 3:** The number of stroke risk factors, early symptoms, and consequences correctly identified by respondents (n = 1013).

Variables		Frequency (%)	Cumulative frequency (%)
Number of correct answers in the general knowledge about stroke	Less than two	83 (8.2%)	83 (8.2%)
	Two	62 (6.1%)	145 (14.3%)
	Three	374 (36.9%)	519 (51.2%)
	Four	299 (29.5%)	818 (80.8%)
	Five	195 (19.2%)	1013 (100.0%)
Number of identified risk factors of stroke	Zero	17 (1.7%)	17 (1.7%)
	One	9 (0.9%)	26 (2.6%)
	Two	15 (1.5%)	41 (4.0%)
	Three	228 (22.5%)	269 (26.6%)
	Four	95 (9.4%)	364 (35.9%)
	Five	96 (9.5%)	460 (45.4%)
	Six	103 (10.2%)	563 (55.6%)
	Seven	75 (7.4%)	638 (63.0%)
	Eight	59 (5.8%)	697 (68.8%)
	Nine	70 (6.9)	767 (75.7%)
	Ten	246 (24.3%)	1013 (100%)
Number of identified early symptoms of stroke	Zero	21 (2.1%)	21 (2.1%)
	One	9 (0.9%)	30 (3.0%)
	Two	27 (2.7%)	57 (5.6%)
	Three	51 (5.0%)	108 (10.7%)
	Four	144 (14.2%)	252 (24.9%)
	Five	288 (28.4%)	540 (53.3%)
	Six	218 (21.5%)	758 (74.8%)
	Seven	255 (25.2%)	1013 (100%)
Number of identified consequences of stroke	Zero	14 (1.4%)	14 (1.4%)
	One	17 (1.7%)	31 (3.1%)
	Two	119 (11.7%)	150 (14.8%)
	Three	285 (28.1%)	435 (42.9%)
	Four	256 (25.3%)	691 (68.2%)
	Five	322 (31.8%)	1013 (100%)

**Figure 1:**
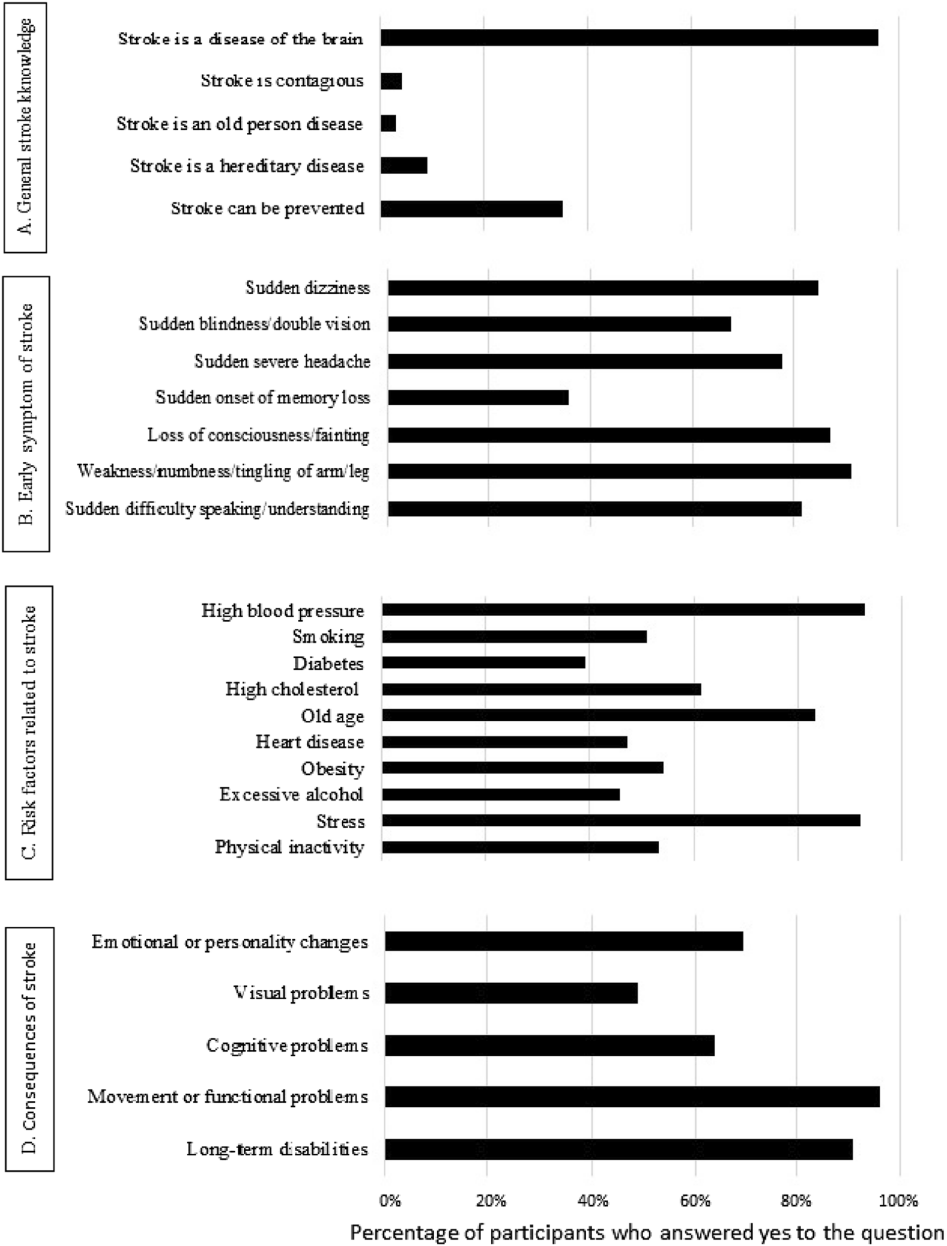
Knowledge of the participants (n = 1013) about (**A**) stroke, (**B**) early symptoms of stroke, (**C**) risk factors related to stroke, and (**D**) consequences of stroke.

Most participants (96.1%) were able to identify the brain as the organ affected by the stroke, however, only (35.1%) were aware that stroke is a preventable disease (Fig. 1A). As for the early symptoms, “Sudden weakness/numbness/tingling of arm/leg” was the most reported warning sign of stroke (91.0%) followed by “Loss of consciousness/fainting” (87.1%) and “Sudden dizziness” (84.7%) (Fig. 1B). High blood pressure (92.8%) followed by stress (92%) and old age (83.3%) were the most common risk factors cited by the respondents while diabetes was the least recognized risk factor (39.2%) (Fig. 1C). Most participants (95.8%) and (90.8%) reported that stroke might lead to functional/movement problem and long-term disabilities, respectively (Fig. 1D).

**Figure 2:**
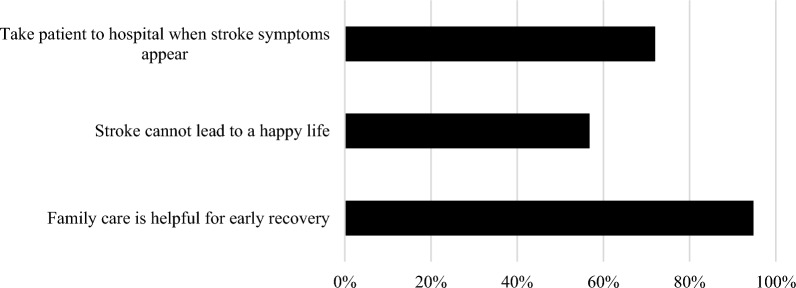
Respondents' attitude and reactions toward stroke (n = 1013).

In the event of witnessing someone with stroke symptoms, the majority (72.0%) reacted by taking the patient to the hospital directly (Fig. 2). In addition, most of the participants (94.8%) reported that family care is helpful for early recovery.

### Sources of the stroke information

**Figure 3:**
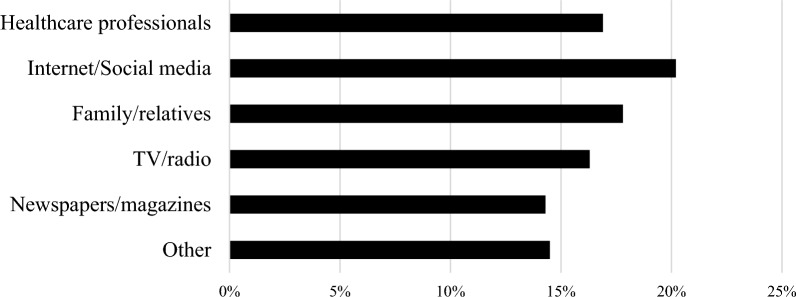
Sources of information about stroke as reported by respondents (n = 1013).

Figure 3 shows the sources of the stroke information mentioned by respondents were internet/social media (20.2%), family/relatives (17.8%), and healthcare professionals (16.9%).

### Bivariate analysis associated with stroke knowledge

Respondents’ recognition of stroke’s early symptoms, risk factors, and consequences are displayed in Table 4. Regarding stroke risk factors, participants who were employed were able to significantly identify at least one correct risk factor compared to those who were unemployed (56.0% versus 44.0%; *p* = 0.029). Also, respondents who were single compared to the other marital status groups (41.7% versus 28.1%, 12.3%, and 17.9%; *p* = 0.036) and smokers compared to non-smokers (67.8% versus 32.2%; *p* < 0.001) were able to correctly recognize at least one stroke risk factor (Table 4).

**Table 4:** Association of risk factors, early symptoms, and consequences of stroke with the socio-demographic characteristics and past medical history of respondents (n = 1013).

Variables		Risk factor(s) identified (≥ 1)			Early symptom(s) identified (≥ 1)			Consequence(s) identified (≥ 1)		
		Yes (n = 996) n (%)	No (n = 17) n (%)	*p* value	Yes (n = 992) n (%)	No (n = 21) n (%)	*p* value	Yes (n = 999) n (%)	No (n = 14) n (%)	*p* value
Socio-demographic characteristics										
Gender	Male	463 (46.5)	8 (47.1)	0.963	465 (46.9)	6 (28.6)	0.096	465 (46.5)	6 (42.9)	0.783
	Female	533 (53.3)	9 (52.9)		527 (53.1)	15 (71.4)		534 (53.5)	8 (57.1)	
Age (years)	< 30	381 (38.3)	11 (64.7)	0.080	381 (38.4)	11 (52.4)	0.371	382 (38.2)	10 (71.4)	**0.025**
	30–49	366 (36.7)	3 (17.6)		364 (36.7)	5 (23.8)		368 (36.8)	1 (7.1)	
	> 50	249 (25.0)	3 (17.6)		247 (24.9)	5 (23.8)		249 (24.9)	3 (21.4)	
Residence area	Urban	785 (78.8)	13 (76.5)	0.768	782 (78.8)	16 (76.2)	0.788	789 (79.0)	9 (64.3)	0.191
	Rural	211 (21.2)	4 (23.5)		210 (21.2)	5 (23.8)		210 (21.0)	5 (35.7)	
Marital status	Single	415 (41.7)	12 (70.6)	**0.036**	417 (42.0)	10 (47.6)	0.864	419 (41.9)	8 (57.1)	0.283
	Married	280 (28.1)	2 (11.8)		276 (27.8)	6 (28.6)		279 (27.9)	3 (21.4)	
	Divorced	123 (12.3)	3 (17.6)		123 (12.4)	3 (14.3)		124 (12.4)	2 (14.3)	
	Widowed	178 (17.9)	0		176 (17.7)	2 (9.5)		177 (17.7)	1 (7.1)	
Educational level	School	347 (34.8)	8 (47.1)	0.295	344 (34.7)	11 (52.4)	0.092	349 (34.9)	6 (42.9)	0.578
	University	649 (65.2)	9 (52.9)		648 (65.3)	10 (47.6)		650 (65.1)	8 (57.1)	
Employment status	Unemployed	438 (44.0)	12 (70.6)	**0.029**	438 (44.2)	12 (57.1)	0.326	442 (44.2)	8 (57.1)	0.335
	Employed	558 (56.0)	5 (29.4)		554 (55.8)	9 (42.9)		557 (55.8)	6 (42.9)	
Income level	Low	622 (62.4)	9 (52.9)	0.684	618 (62.3)	13 (61.9)	0.436	625 (62.6)	6 (42.9)	0.105
	Medium	221 (22.2)	5 (29.4)		223 (22.5)	3 (14.3)		222 (22.2)	4 (28.6)	
	High	153 (15.4)	3 (17.6)		151 (15.2)	5 (23.8)		152 (15.2)	4 (28.6)	
Smoking status	No	321 (32.2)	13 (76.5)	** < 0.001**	319 (32.2)	15 (71.4)	** < 0.001**	325 (32.5)	9 (64.3)	**0.019**
	Yes	675 (67.8)	4 (23.5)		673 (67.8)	6 (28.6)		674 (67.5)	5 (35.7)	
Past medical history										
Hypertension	No	715 (71.8)	11 (64.7)	0.588	710 (71.6)	16 (76.2)	0.642	714 (71.5)	12 (85.7)	0.372
	Yes	281 (28.2)	6 (35.3)		282 (28.4)	5 (28.4)		285 (28.5)	2 (14.3)	
Diabetes	No	900 (90.4)	16 (94.1)	1.000	897 (90.4)	19 (90.5)	1.000	902 (90.3)	14 (100)	0.385
	Yes	96 (9.6)	1 (5.9)		95 (9.6)	2 (9.5)		97 (9.7)	0	
Dyslipidemia	No	785 (78.8)	13 (76.5)	0.768	779 (78.5)	19 (90.5)	0.280	784 (78.5)	14 (100)	0.050
	Yes	211 (21.2)	4 (23.5)		213 (21.5)	2 (9.5)		215 (21.5)	0	
Arrhythmia	No	797 (80.0)	15 (88.2)	0.548	794 (80.0)	18 (85.7)	0.782	800 (80.1)	12 (85.7)	1.000
	Yes	199 (20.0)	2 (11.8)		198 (20.0)	3 (14.3)		199 (19.9)	2 (14.3)	
Kidney disease	No	913 (91.7)	14 (82.4)	0.169	907 (91.4)	20 (95.2)	1.000	913 (91.4)	14 (100)	0.623
	Yes	83 (8.3)	3 (17.6)		85 (8.6)	1 (4.8)		86 (8.6)	0	
Depression	No	839 (84.2)	12 (70.6)	0.171	835 (84.2)	16 (76.2)	0.361	838 (83.9)	13 (92.9)	0.711
	Yes	157 (15.8)	5 (29.4)		157 (15.8)	5 (23.8)		161 (16.1)	1 (7.1)	
Obesity	No	900 (90.4)	13 (76.5)	0.078	897 (90.4)	16 (76.2)	0.048	899 (90)	14 (100)	0.384
	Yes	96 (9.6)	4 (23.5)		95 (9.6)	5 (23.8)		100 (10)	0	

Numbers in bold indicate significant *p*-values.

Chi-squared test was done to preform the analysis.

For the early symptoms, smokers as compared to non-smokers (67.8% versus 32.2%; *p* < 0.001) and those with no history of obesity compared to participants who had obesity (90.4% versus 9.6%; *p* = 0.048) were significantly more likely to identify at least one correct stroke symptom (Table 4).

For the consequences, respondents of the younger age group (below 30 years) were significantly able to list at least one correct stroke consequence compared to the older age groups (38.2% versus 36.8% and 24.9%; *p* = 0.025). Moreover, a significantly higher number of smokers compared to non-smokers correctly identified at least one stroke consequence (67.5% versus 32.5%; *p* = 0.019).

### Bivariate analysis associated with response to somebody with symptoms of stroke

Around three quarters of the participants (72.0%) reported that their first action in response to witnessing a patient with stroke symptoms was to take the patient directly to a hospital. Table 5 shows association of respondents’ reactions by taking a patient who is experiencing a stroke to the hospital with socio-demographic characteristics, and past medical history. Significant relationships (*p* < 0.05) were found between most socio-demographic characteristics, and past medical history and respondents’ reactions by taking a patient who is experiencing a stroke to the hospital.

**Table 5:** Association of respondents’ reactions by taking a patient who is experiencing a stroke to the hospital with socio-demographic characteristics, and past medical history (n = 1013).

Variables		Taking a patient who is experiencing a stroke to the hospital		
		Yes (n = 729), n(%)	No (n = 284), n(%)	p value
Socio-demographic characteristics				
Gender	Male	359 (49.2)	112 (39.4)	**0.005**
	Female	370 (50.8)	172 (60.6)	
Age (years)	< 30	343 (47.1)	49 (17.3)	**< 0.001**
	30–49	328 (45.0)	41 (14.4)	
	> 50	58 (8.0)	194 (68.3)	
Residence area	Urban	593 (81.3)	205 (72.2)	**0.001**
	Rural	136 (18.7)	79 (27.8)	
Marital status	Single	382 (52.4)	45 (15.8)	**< 0.001**
	Married	236 (32.4)	46 (16.2)	
	Divorced	64 (8.8)	62 (21.8)	
	Widowed	47 (6.4)	131 (46.1)	
Educational level	School	154 (21.1)	201 (70.8)	**< 0.001**
	University	575 (78.9)	83 (29.2)	
Employment status	Unemployed	266 (36.5)	184 (64.8)	**< 0.001**
	Employed	463 (63.5)	100 (35.2)	
Income level	Low	400 (54.9)	231 (81.3)	**< 0.001**
	Medium	196 (26.9)	30 (10.6)	
	High	133 (18.2)	23 (8.1)	
Smoking	No	260 (35.7)	74 (26.1)	**0.003**
	Yes	469 (64.3)	210 (73.9)	
Past medical history				
Hypertension	No	627 (86.0)	99 (34.9)	**< 0.001**
	Yes	102 (14.0)	185 (65.1)	
Diabetes mellitus	No	692 (94.9)	224 (78.9)	**< 0.001**
	Yes	37 (5.1)	60 (21.1)	
Dyslipidemia	No	666 (91.4)	132 (46.5)	**< 0.001**
	Yes	63 (8.6)	152 (53.5)	
Arrhythmia	No	651 (89.3)	161 (56.7)	**< 0.001**
	Yes	78 (10.7)	123 (43.3)	
Kidney disease	No	683 (93.7)	244 (85.9)	**< 0.001**
	Yes	46 (6.3)	40 (14.1)	
Depression	No	654 (89.7)	197 (69.4)	**< 0.001**
	Yes	75 (10.3)	87 (30.6)	
Obesity	No	656 (90.0)	257 (90.5)	0.808
	Yes	73 (10.0)	27 (9.5)	

Numbers in bold indicate significant p values.

### Multivariable analysis associated with stroke knowledge

In the multiple logistic regression analysis, being a smoker compared to a non-smoker (OR 6.117, *p* = 0.004) was associated with better identification of at least one stroke risk factor (Table 6). In addition, having a higher educational level-university compared to school level- (OR 3.207, *p* = 0.011) and being a smoker compared to a non-smoker (OR 6.915, *p* < 0.001) were significantly associated with higher odds of early stroke symptoms identification. As for the stroke consequences, respondents in the age group (30–49 years) were more likely to identify at least one stroke consequence as compared to the younger age group (below 30 years) (OR 9.229, *p* = 0.035). Besides, participants who were females, above 50 years, and those with lower educational level were more likely to consider taking a patient experiencing stroke symptoms to the hospital (*p* < 0.001). Moreover, having a medical history of hypertension and depression compared to no history was associated with significantly higher odds of responding appropriately to a patient with acute stroke symptoms by taking to the hospital (*p*-values of 0.002 and 0.005, respectively).

**Table 6:** Multivariable analysis (n = 1013).

Variables	β (SE)	OR (95% CI)	*p* value
Risk factor(s) identified (≥ 1)			
Employment status (employed versus unemployed*)	0.950 (0.564)	2.585 (0.856–7.801)	0.092
Smoker (yes versus no*)	1.811 (0.625)	6.117 (1.795–20.838)	**0.004**
Early symptom(s) identified (≥ 1)			
Educational level (university versus school*)	1.165 (0.459)	3.207 (1.304–7.888)	**0.011**
Smoker (yes versus no*)	1.934 (0.503)	6.915 (2.581–18.525)	** < 0.001**
Consequence(s) identified (≥ 1)			
Age group (30–49 versus < 30 years*)	2.222 (1.052)	9.229 (1.175–72.479)	**0.035**
Age group (> 50 versus < 30 years*)	-0.236 (0.669)	0.790 (0.213–2.931)	0.724
Taking a patient to a hospital			
Gender (females versus males*)	0.747 (0.206)	2.111 (1.409–3.161)	** < 0.001**
Age (> 50 versus < 30 years*)	2.099 (0.257)	8.158 (4.926–13.509)	** < 0.001**
Educational level (university versus school level*)	-1.219 (0.210)	0.296 (0.196–0.446)	** < 0.001**
Hypertension (yes versus no*)	0.762 (0.250)	2.143 (1.314–3.497)	**0.002**
Depression (yes versus no*)	0.741 (0.268)	2.098 (1.247–3.528)	**0.005**

Bold values represent significant results.

*: stands for the referencce category.

*β* Beta, *SE* standard error, *OR* adjusted ratio, *CI* confidence interval.

Logistic regression taking identification of stroke risk factors, stroke early symptoms, stroke consequences, taking a patient who is experiencing stroke to the hospital as the dependent variables and sociodemographic factors (gender, residence area, educational level, employment status, and smoking history) and past medical history as independent variables.

## Discussion

The present study aimed at examining factors associated with stroke knowledge and awareness among the general population of Syria, by factoring in the familiarity of participants with stroke, its associated risk factors, early symptoms, consequences, and participants’ reactions upon encountering a patient with a stroke. More than half of the sample were females, employed, had low monthly income, educated with university certificate and smokers. In addition, more than one-third of the participants were below 30 years and single. From a total of 1013 respondents, almost everyone who took part in the study, had heard of the condition previously and reported that stroke is a disease of brain. However, when the participants’ general knowledge on stroke was assessed, less than a quarter of the participants were correctly able to identify all the risk factors, able to identify all the consequences, and symptoms of stroke.

Taking into account recent studies in the region to contextualize our findings, in Jordan (2022; n = 573)^19^, Lebanon (2022; n = 551)^17^, Iraq (2023; n = 606)^31^, Saudia Arabia (2023; n = 389)^32^, and the UAE (2023; n = 545)^30^, and Sudan (2024; n = 410)^18^ awareness that stroke is a type of brain disease was various among the participants with the highest in Jordan and Lebanon with more than 95.0% of the participants, then Iraq (92.8%), Sudan (02.2%), Saudia Arabia (89.7%), and the UAE (70.8%), while the results of our studies indicated that 96.1% of the participants were able to identify the brain as the organ affected by the stroke. In related to the participants’ awareness of stroke is preventable, the results reported by the participants were also various among the countries, with the highest in Iraq (85.6%)^31^, then Sudan (83.4%)^18^, Jordan and Saudia Arabia with 81.0%^19,32^, Lebanon (80.0%)^17^, and the UAE (42.9%)^30^, while less percentage with 35.1% in our study participants were aware of this. This reveals that our study population exhibits relatively weak understanding of the preventable nature of the disease. It could be plausible that since (65%) of our respondents were educated till the university level. Factually, according to the World Stroke Organization (WSO), up to (90%) of strokes may be prevented even if a few modifiable risk factors, such as hypertension, diet, smoking, and exercise, are addressed^34^.

High blood pressure was the most acclaimed risk factor for stroke by our respondents (92.8%) and respondents of the Jordan study (92.1%), Iraq study (91.1%), Sudan study (90.2%) and the UAE study (91.0%)^18,19,30,31^, whereas psychological stress was stated by most of the Lebanon respondents (90.0%) and Saudi Arabia respondents (80.0%)^17,32^. Nonetheless, the single most important risk factor for stroke is high blood pressure^35,36^. Other risk factors^37,38^, however, do include psychosocial stress, depression, diabetes mellitus, impulsive alcohol intake, and cardiac illnesses, among others.

Furthermore, a remarkable number of our participants (60.8%) felt that diabetes is not a risk factor of stroke. Such a finding is indifferent, since previous studies have also reported that diabetes is identified less as a risk factor among its participants^19,34,37,38^. This showed the gap in knowledge that should be addressed, as it is evident through multiple studies that diabetes is associated with stroke^39–41^.

Our study findings also indicated the highest percentage of participants were able to identify at least one risk factor of stroke (98.3%) when compared with participants from similar studies, such as those reported by Sug Yoon et al. in Australia (76.2%; n = 822)^8^, Barakat et al. in Jordan (98.1%; n = 573)^19^, Malaeb et al. in Lebanon (97.8%; n = 551)^17^, Khalil and Lahoud in Lebanon (85.4%; n = 390)^20^, Sundseth et al. in Norway (43.2%; n = 124)^42^, Duque et al. in Portugal (84.3%; n = 252)^43^, in AlObaidi et al. in Iraq (85.6%; n = 609)^31^, Alhazzani et al. in Saudi Arabia (78.7%; n = 1472)^44^, Alzayer et al. in Saudi Arabia (99.5%; n = 389)^32^, Jirjees et al. in UAE (99.8%; n = 545)^30^, Eltayib etal. in Sudan (96.3%; n=410)^18^ Segura et al. in Spain (59.6%, n = 2884)^45^, and Kaddumukasa et al. in Uganda (73.4%; n = 377)^46^.

Moreover, respondents who were employed, single and smokers were able to significantly identify at least risk factor of stroke than others. As evident by multiple studies, employment status undoubtedly impacts health literacy levels^47–51^^56^. This suggests that it may be crucial to consider the workplace as a setting for establishing health literacy skills. Although basic literacy skills are necessary for health literacy but accessing and utilizing health information is also dependent on an individual's capacity for action, which is a crucial component of health promotion^52^. Additionally, people who are not employed miss out on workplace environments that promote health literacy^47^. Furthermore, single participants as well as smokers significantly identified at least one risk factor. Such results could be seen since our cohort of respondents were mainly single (42.2%), followed by those who were married (27.8%), divorced (12.4%) and widowed (17.6%), and that 67% were smokers. Same goes for the results seen with regard to significant association between smokers and at least one identification of early stroke symptoms and stroke consequences.

Our multivariable analysis revealed that being educated at the university level was significantly related with higher chances of identifying early stroke symptoms (OR 3.207) which is consistent with the study conducted among Jordanian population (OR 3.4)^19^, indicating that education is paramount in being health literate. Our findings can be interpreted by the fact that educated people have higher levels of health literacy, access the medical information resources easily, and have ability to interpret health information which all enhance the level of knowledge about diseases in general and stroke in particular. Whereas non-smokers in our study were correlated with early symptoms identification. Interestingly, although the primary sources of information regarding stroke was the internet/social media in all three studies of Syria, Jordan, and Lebanon 20.2%, 24.4% and 30% respectively, health-care providers were contacted the least in Syria (16.9%) than in Lebanon (30%) and in Jordan (20.9%)^17,19^.

Although certain studies have indicated a higher likelihood of poor health literacy among smokers^53,54^, other research has contradicted this finding^55,56^. In our study, we focused on the latter group, as it suggests that being a smoker does not necessarily imply a lack of health literacy.

Furthermore, when participants were questioned about their curiosity for additional information on stroke, an overwhelming majority (93.7%) expressed their interest in gaining further knowledge on the subject. This emphasizes the urgent necessity of educating the Syrian population to improve their health literacy levels, especially in light of the country's literacy reports (last updated in 2004)^57^.

Although the Syrian population exhibits a reasonable level of understanding regarding stroke, it remains insufficient, particularly concerning the awareness that stroke can be prevented and that visual problems may result from it. Surprisingly, over half of the participants believed that visual problems were not a potential consequence of stroke. This fact was further highlighted in a prospective epidemiological study conducted in a multi-center setting, where one of the objectives was to investigate the point prevalence of visual impairments among 1033 acute adult stroke patients. The study revealed a significant point prevalence of visual problems at 73%, with 56% of the patients experiencing impaired central vision^58^.

In light of these findings, it is strongly advised that local health organizations in Syria initiate and maintain continuous stroke awareness campaigns. These campaigns should prioritize educating the population about stroke, its warning signs, and effective prevention strategies. By raising awareness and disseminating crucial information, these efforts can contribute to reducing the incidence of stroke and promoting better overall health outcomes within the Syrian community.

### Strengths and limitations

The level of awareness and understanding of strokes, in Syria has yet to be examined on a scale. Given that Syria's a developing country situated in the Middle East it becomes crucial to assess the gaps in knowledge and the perceptions related to strokes within this region. This study’s objective was to fill this knowledge gap by conducting the ever-nationwide survey aimed at evaluating the Syrian populations level of knowledge and awareness when it comes to strokes. By shedding light on these knowledge gaps and public perceptions this research will provide insights for stroke education and initiatives promoting awareness, in the country.

The study exhibits certain limitations. Firstly, due to its cross-sectional design, the ability to establish causality and association is limited. Thus, the inclusion of additional longitudinal studies is necessary to establish causality and track the changes in awareness over time. Secondly, the utilization of an online distribution method poses challenges in impeding to reach out to the different demographics mainly elderly and individuals from lower socioeconomic classes that may lead to underrepresentation. To address this limitation, a two-stage approach was employed, wherein the questionnaire was also distributed in paper form on-site during the second stage.

Thirdly, it should be acknowledged that this study was conducted within a country grappling with two significant crises, characterized by distinct circumstances. Consequently, the generalizability of the results may be constrained solely to countries sharing a similar situation.

In addition, it should be noted that the generalizability of our findings may be further influenced by the inherent heterogeneity within the selected districts, encompassing diverse socio-cultural contexts, which could impact health awareness levels and contribute to potential variations in the observed outcomes.

## Conclusion

The findings of the study indicated that there is a relatively weak understanding of the preventable nature of stroke among Syrian population. Smokers, employed people, and single Syrians were more capable of identifying at least one risk factor for stroke compared to others. Despite some level of understanding of stroke among the participants, the overall awareness is still inadequate, particularly in terms of knowing that stroke can be prevented and that visual problems can occur as a result of a stroke.

## Data Availability

The datasets generated during and/or analysed during the current study are available from the corresponding author on reasonable request.
